# Recipient and donor PTX3 rs2305619 polymorphisms increase the susceptibility to invasive fungal disease following haploidentical stem cell transplantation: a prospective study

**DOI:** 10.1186/s12879-022-07298-2

**Published:** 2022-03-26

**Authors:** Chen Zhao, Xiao-Su Zhao, Lan-Ping Xu, Xiao-Hui Zhang, Xiao-Jun Huang, Yu-Qian Sun

**Affiliations:** 1 Peking University People’s Hospital, Peking University Institute of Hematology, National Clinical Research Center for Hematologic Disease, 11 Xizhimen South Street, Beijing, 100044 People’s Republic of China; 2 Beijing Key Laboratory of Hematopoietic Stem Cell Transplantation for the Treatment of Hematological Diseases, Beijing, People’s Republic of China; 3 Peking-Tsinghua Center for Life Sciences, Beijing, People’s Republic of China

**Keywords:** PTX3 rs2305619 polymorphism, Invasive fungal disease, Haploidentical stem cell transplantation

## Abstract

**Background:**

Invasive fungal disease (IFD) is a severe complication after haploidentical stem cell transplantation (haplo-HSCT) and has a poor prognosis. It has been shown that genetic polymorphism may be one possible reason for the increased risk of IFD. This study aimed to assess the role of genetic polymorphism in the level of susceptibility to IFD after haplo-HSCT.

**Methods:**

In this study, we prospectively enrolled 251 patients who received haplo-HSCT at the Peking University Institute of Hematology from 2016 to 2018. Forty-three single nucleotide polymorphisms (SNPs) of the genomic DNA were genotyped in blood samples from both recipient and donor.

**Results:**

Twenty-two patients (8.8%) were diagnosed with proven or probable IFD. The independent risk factors for IFD were grades 3–4 acute graft-versus-host disease, cytomegalovirus reactivation, and recipient and donor rs2305619 (PTX3) (P < 0.05) in multivariate analysis. Meanwhile, we combined the variables to develop the IFD risk scoring system and stratified patients into low- (0–2) and high-risk (3–4) groups. The 30-day and 100-day cumulative incidence of IFD in the low- and high-risk groups were 2.1% and 10.2%, 4.2% and 20.3%, respectively (P = 0.015).

**Conclusions:**

PTX3 rs2305619 polymorphism increase the susceptibility of IFD after haplo-HSCT in the Chinese Han population, and the IFD scoring system could be useful in risk stratification for IFD after HSCT.

**Supplementary Information:**

The online version contains supplementary material available at 10.1186/s12879-022-07298-2.

## Introduction

Invasive fungal disease (IFD) is a common and major complication in patients following haploidentical stem cell transplantation (haplo-HSCT), primarily because of the severe immune-compromised status of the host. It has been shown that the incidence of IFD in human leukocyte antigen (HLA)-matched sibling transplantation is about 5–10% and can be as high as 20% in mismatched/haploidentical stem cell transplantation [1–3]. Meanwhile, the overall mortality rate was approximately 30% [4]. Because of its high incidence, difficulty in diagnosis, poor prognosis, and high economic burden, it is very important to prevent the development of IFD.

It has been demonstrated that antifungal prophylaxis can decrease the incidence of IFD and all-cause mortality in allogeneic stem cell transplantation recipients [5]. However, it was suggested that for those with a low risk of IFD, antifungal prophylaxis does not lead to any survival benefit and may also lead to significant burden due to medical costs and organ toxicity. Therefore, a risk-based antifungal prophylaxis strategy was proposed.

The well-known risk factors of IFD following stem cell transplantation include advanced disease stage at transplant, prior IFD history before transplantation, diabetes, haploidentical donors, prolonged severe neutropenia, severe graft-versus-host disease (GVHD), and cytomegalovirus (CMV) [3, 6]. However, all of the risk factors mentioned above were assessed based on clinical variables. Even under the same clinical conditions, there are significant discrepancies in the risk of IFD among different patients. Furthermore, patients with no significant clinical risk factors might still have complications associated with IFD. In brief, a risk model based only on clinical variables is not sufficient to predict IFD.

It has been noticed that the host immune system has an important impact on susceptibility to IFD. Several steps may be involved in the process of antifungal immunity, including recognition, adaptive immunity, and phagocytosis. Therefore, in addition to the above clinical conditions, there may be other factors that lead to differences in susceptibility to IFD, which may be related to individual differences in the host’s immune response. It has been shown that there is an association between the single nucleotide polymorphisms (SNPs) of the host innate immune system and IFD susceptibility, namely that SNPs affect the development of IFD [7, 8]. Many studies have reported that genetic deficiencies in Dectin-1, pentraxin-3 (PTX3), TLR2, and TLR4 confer high susceptibility to IFD after hematopoietic stem cell transplantation (HSCT) [9–12]. However, research on the association between SNPs and IFD has been limited to Caucasians, and the association remains unclear in the Chinese Han population. Furthermore, there is no such study in the setting of haplo-HSCT. This study aimed to assess the role of genetic polymorphism in susceptibility to IFD following haplo-HSCT in the Chinese Han population. The goal of this study was to develop a risk scoring system for IFD.

## Methods

### Patients

Consecutive patients who received haplo-HSCT between June 2016 and December 2018 at the Peking University Institute of Hematology, Peking University People’s Hospital, were prospectively recruited if they fulfilled the following criteria: first haploidentical stem cell transplantation, donor and recipient were both from the Chinese Han population, no previous IFD history, and not before HSCT. The Ethics Committee of Peking University People’s Hospital approved this study. All procedures involving human participants were performed in accordance with the ethical standards of the institutional and/or national research committee, the Declaration of Helsinki, or comparable ethical standards. All patients provided written informed consent to participate in the study.

### Haploidentical stem cell transplantation protocol

The principles of human leukocyte antigen typing, donor selection, donor stem cell harvesting, conditioning, and prevention of graft-versus-host disease (GVHD) and infection have been described in a previous report [13]. All recipients of transplants from haploidentical donors received a uniform modified busulfan/cyclophosphamide/anti-thymocyte globulin conditioning regimen, which consisted of cytarabine (4 g/m^2^/day on days − 10 to − 9), busulfan (3.2 mg/kg/day, administered intravenously on days − 8 to − 6), cyclophosphamide (1.8 g/m^2^/day on days − 5 to − 4), semustine (250 mg/m^2^, administered orally on day − 3), and rabbit anti-thymocyte globulin (2.5 mg/kg/day [Sang Stat, Lyon, France] on days − 5 to − 2). Cyclosporine plus short-term methotrexate and mycophenolate mofetil were administered to prevent GVHD. Methotrexate was administered intravenously on day one (15 mg/m^2^) and then again (10 mg/m^2^) on days 3, 6, and 11 after transplantation.

### Management of IFD

Prophylaxis was based on triazoles, specifically fluconazole and itraconazole, in primary prevention. Prophylaxis stops at 75 days after HSCT if patients do not develop IFD. Patients were prospectively monitored with β-(1–3)-glucans (G)/galactomannans (GM) twice weekly. A CT scan was performed after engraftment and any time there was clinical suspicion of IFD. IFD was categorized as proven, probable, or possible according to the European Organization for Research and Treatment of Cancer and the Mycoses Study Group and Research Consortium (EORTC/MSG) 2019 criteria [14]. Patients were diagnosed as having suspected IFD if they had IFD risk factors, were observed to have symptoms, radiological abnormalities, or indirect microbiological evidence of fungal infection, and were treated empirically with antifungal agents. Treatment practices were determined by treating physicians according to their usual practice and judgment. Patients were followed up for at least 1 year after the date of transplantation, and follow-up was completed on December 30, 2019. Documentation continued until the completion of observation or death.

### Definitions

The primary endpoint was the 1-year cumulative incidence of IFD. IFD was diagnosed according to the EORTC/MSG 2019 criteria. This study included patients with proven or probable IFD. Neutrophil engraftment was defined as the first of 3 consecutive days with an absolute neutrophil count ≥ 0.5 × 10^9^/L. Platelet engraftment was defined as the first of 7 consecutive days with a platelet count ≥ 20 × 10^9^/L without transfusion dependence, and complete donor chimerism was defined as the presence of at least 95% donor hematopoietic cells. Primary graft failure was defined as the failure to surpass a threshold absolute neutrophil count of 0.5 × 10^9^/L by day 28 after transplantation. Acute graft-versus-host disease (aGVHD) and chronic GVHD (cGVHD) were graded according to previous criteria [15]. Overall survival (OS) was defined as the time from the first day of transplantation to death as a result of any cause. Follow-up data for survival were censored when the patient was last verified to be alive. Disease-free survival (DFS) was defined as the time from transplantation to relapse, disease progression, or death, whichever occurred first. Treatment-related mortality (TRM) was defined as any cause of death other than relapse. Relapse was defined as the reappearance of blasts in the blood, BM (> 5%), or any extramedullary site after complete response (CR).

### Collection of DNA samples and SNP typing

Peripheral blood (4 mL) with ethylenediaminetetraacetic acid (EDTA) was collected from both the recipients and the donors, and peripheral blood mononuclear cells (PBMCs) were isolated by density gradient centrifugation [16]. Genomic DNA was extracted from PBMCs using the QIAamp DNA Blood Mini Kit. The SNPs were determined using real-time polymerase chain reaction (RT-PCR) assays. In total, 21 pattern recognition receptors (PRRs) and cytokines, chemokines, whose 43 SNPs of TLR4, TLR1, TLR3, TLR6, TLR5, TLR9, DC-SIGN, PTX3, Dectin-1, MBL2, IFN-γ, TNF-α, IL-10, IL-4, IL-17A, IL-17F, IL-1RN, IL-1B, IL-6, IL-23, and C-X-C-10 were detected based on their previously published association with IFD (Additional file 1: Table S1). The samples were used to assess the association between susceptibility to IFD and SNPs.

### Statistics analysis

Continuous variables were presented as the median, and categorical variables were presented as percentages. OS and DFS were estimated using the Kaplan–Meier method. The cumulative incidences of engraftment, GVHD, TRM, and relapse were estimated using the competing risks model. Death and relapse without developing GVHD were treated as competing events for GVHD, whereas relapse and NRM were treated as events competing with each other. A p-value < 0.1 for a two-sided test was considered to be significant. CMV reactivation and aGVHD were treated as time dependent variables, therefore only those occurring before IFD were included in analysis. The multivariate Cox proportional model and survival analysis were calculated with SPSS software (SPSS 16.0, Chicago, IL, USA). The cumulative incidence was calculated with R statistical software, version 3.6.0 (R Foundation for Statistical Computing, Vienna, Austria). Data were analyzed with the Statistical Package for Social Sciences (SPSS) software, revision 20.0. SNPs with a value of P < 0.1 in the analysis of the association with susceptibility to infection were included as confounders in the multiple Cox regression. P values less than 0.05 were considered statistically significant. The hazard ratio (HR) and their 95% confidence intervals (CIs) were calculated. To determine risk factors associated with the occurrence of IFD, variables including recipient age, gender, underlying disease, donor age, gender, donor–recipient ABO blood type, neutrophil engraftment, platelet engraftment, acute GVHD, chronic GVHD, CMV reactivation, Epstein–Barr virus, and SNPs of recipients and donors were included in univariate analysis. Variables with p-values < 0.10 in univariate models were entered into Multivariate Cox regression models. The score system was built by factors (grades 3–4 aGVHD, CMV reactivation, recipient rs2305619 and donor rs2305619) identified in multivariate analysis, and each factor was assigned 1 score.

## Results

### Demographics

A total of 251 pairs of recipients and donors were enrolled in this study, all of whom received haploidentical transplantation. Their characteristics are summarized in Table 1. Overall, there were 140 (55.8%) males and 111 (44.2%) females with a median age of 28 (2–63) years. Among the patients, 70 (27.9%) were children, and 181 (72.1%) were adults. Among the donors, 190 (75.7%) were males, and 61 (24.3%) were females, with a median age of 36 (4–64) years. The median doses of infused mononuclear cells and CD34+ cells were 8.74 (6.16–13.97) × 10^8^/kg recipient body weight and 2.76 (0.68–6.03) × 10^6^/kg recipient body weight, respectively.

**Table 1:** Demographic and clinical characteristics of recipients and donors

Variables	Total number (n = 251)
Recipient age (y), median	28 (2–63)
Children (**≤** 16)	70 (27.9%)
Adults (**>** 16)	181 (72.1%)
Male sex, *n* (%)	140 (55.8%)
Underling disease (%)	
AML	102 (40.6%)
ALL	99 (39.4%)
AA	20 (8.0%)
MDS	19 (7.6%)
Others	11 (4.4%)
Donor age (y), median	36 (4–64)
Male donor sex, n (%)	190 (75.7%)
Donor–recipient ABO blood type	
Match, *n* (%)	138 (55.0%)
Major mismatch, *n* (%)	53 (21.1%)
Minor mismatch, *n* (%)	60 (23.9%)
Mononuclear cells (× 10^8^/kg)	8.74 (6.16–13.97)
CD34^+^ cells (× 10^6^/kg)	2.76 (0.68–6.03)
IFD prophylaxis	
Fluconazole	9 (3.6%)
Itraconazole	181 (72.1%)
Voriconazole	53 (21.1%)
Caspofungin	8 (3.2%)

*AML* acute myeloid leukemia, *ALL* acute lympphocytic leukemia, *AA* aplastic anemia, *MDS* myelodysplastic syndrome, *IFD* invasive fungal disease

### Transplantation outcome of the overall cohort

#### Engraftment

Three patients died early (within 28 days after transplantation) were not applicable to evaluate engraftment, while the 248 remaining patients all achieved neutrophil engraftment, with a median time of 15 (9–46) days after transplantation. In addition, 235 out of 251 patients (93.6%) achieved successful platelet engraftment, with a median time of 18 (7–237) days after transplantation.

#### GVHD

**Fig. 1:**
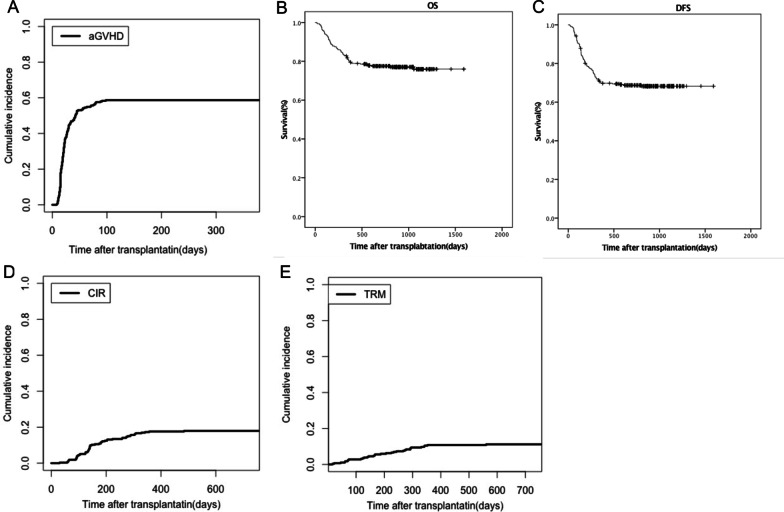
Outcomes of 251 patients after transplantation. **A** The cumulative incidence of acute graft-versus-host disease (aGVHD) curves after transplantation; **B** overall survival (OS) and **C** disease-free survival (DFS) in patients who received HSCT; **D** relapse curves and **E** treatment-related mortality (TRM) curves after HSCT

A total of 180 patients (71.7%) developed acute GVHD, and 154 (61.4%) were grades I–II, while 26 (10.4%) were grades III–IV. The median time from transplantation to onset of aGVHD was 28 (8–98) days. The cumulative incidence of aGVDH at 30 days and 100 days was 51.9% (95% CI 34.5–69.3%) and 68.7% (95% CI 53.6–83.8%), respectively (Fig. 1A). In the overall population, 63 patients (25.1%) developed chronic GVHD, with a median time of 201 (104–730) days, and the 1-year cumulative incidence was 22.4% (95% CI 22.3–22.5%).

#### Survival

The median follow-up time was 786 (16–1592) days. There were 58 deaths during this study, resulting in an overall mortality rate of 23.1% (58/251), and 12 deaths (54.5%) in IFD patients. The 2-year OS was 77.5% (95% CI 73.1–82.2%; Fig. 1B), and DFS was 68.7% (95% CI 63.9–73.5%; Fig. 1C).

#### Relapse

In all, 45 (20.9%) patients relapsed at a median time of 223 (30–1014) days post-transplantation. The 2-year cumulative incidence of relapse (CIR) was 17.5% (95% CI 16.7–18.3%; Fig. 1D).

#### Treatment-related mortality

There were 33 (13.1%) patients who died due to treatment-related mortality (TRM), and the 2-year cumulative TRM was 14.5% (95% CI 13.9–15.1%; Fig. 1E).

### The characteristics of IFD

A total of 22 patients (8.8%) diagnosed with IFD are shown in Table 2. The incidence of proven and probable IFD was 2.79% (7/251) and 5.98% (15/251), respectively. The 6-month cumulative incidence of IFD was 7.96 (95% CI 7.49–8.43%), and the 1-year cumulative incidence was 8.30% (95% CI 7.81–8.79%). The median onset time was 59 (9–262) days after transplantation. Of the all IFD patients, nine developed IFD within 30 days after transplantation, 12 developed it within 6 months (41–170 days), and one occurred later than 6 months (262 days) after transplantation. In the proven/probable IFD cases, 15 (68.2%) developed in the respiratory tract, three (13.6%) in blood, three (13.6%) in the central nervous system, and one (4.5%) in the digestive tract. The causal fungal species was identified in only eight patients, including Aspergillus (2/8, 25.0%), Candida (3/8, 37.5%), and non-Aspergillus molds infections [3/8, 37.5% (including one Rhizopus, one Mucorales, and one *Absidia orchidis* infection)].

**Table 2:** Characteristics of IFD

Variables	
Cumulative incidence	
100 d	6.97% (95% CI 6.56–7.38%)
6 Mo	7.96% (95% CI 7.49–8.43%)
1 year	8.30% (95% CI 7.81–8.79%)
IFD diagnosis levels	
Proven	7 (2.79%)
Probable	15 (5.98%)
The median time of IFD	59 (9–262) days
Fungal species	
*Aspergillus*	2/8 (25.0%)
*Candida*	3/8 (37.5%)
*Rhizopus*	1/8 (12.5%)
*Mucorales*	1/8(12.5%)
*Absidia orchidis*	1/8 (12.5%)
Site of fungal infection	
Respiratory tract	15 (68.2%)
Central nervous system	3 (13.6%)
Blood	3 (13.6%)
Digestive tract	1 (4.5%)

*IFD* invasive fungal disease

**Fig. 2:**
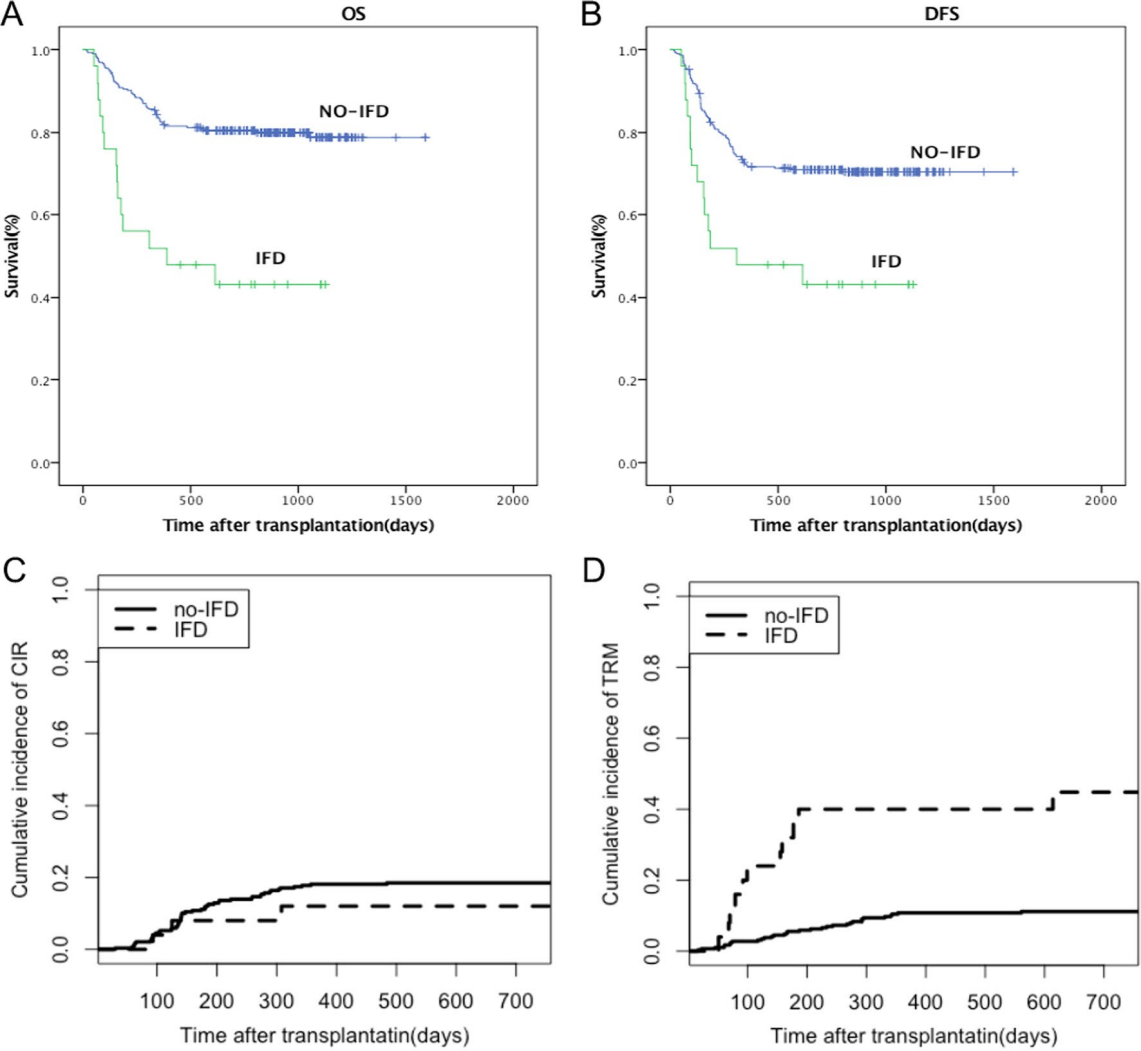
The impact of invasive fungal disease (IFD) on transplantation outcome. **A** OS and **B** DFS in IFD patients, compared to no-IFD patients after transplantation; **C** relapse and **D** TRM in IFD patients, compared to no-IFD patients after transplantation

Eleven IFD patients died. Of the 11 patients, eight died due to infection, which included three who died due to serious fungal infection, one due to diffuse alveolar hemorrhage, and two due to multiple organ failure. Compared with patients without IFD, patients with IFD experienced poor overall survival, with a 1-year OS of 52.0% (95% CI 32.4–71.6%) vs. 82.6% (95% CI 78.7–86.5%; P < 0.01; Fig. 2A). The 1-year DFS were 48.0% (95% CI 28.4–49.96%) for IFD patients and 71.7% (95% CI 57.4–86.0%) for no-IFD patients, respectively (P < 0.01; Fig. 2B). Among the IFD patients, three relapsed at a median time of 175 (93–308) days. The 1-year CIR was 12.0% (95% CI 11.2–12.8%), while without IFD, the 1-year CIR was 18.1% (95% CI 18.0–18.2%; P = 0.42; Fig. 2C). The 1-year cumulative incidence of TRM in IFD and no-IFD patients was 40.0% (95% CI 38.1–41.9%) and 10.8 (95% CI 10.7–10.9%), respectively (P < 0.01; Fig. 2D).

### Risk factors of IFD

Risk factors for IFD among clinical characteristics of transplant recipients and polymorphism of recipients and donors are presented in Table 3. There were no significant differences in pre-transplant variables including gender, underlying disease, and donor–recipient ABO blood type or post-transplant variables including neutrophil engraftment, chronic GVHD, and Epstein–Barr virus. In the univariate analysis, recipient age (> 16 years), CMV reactivation, acute GVHD (grades 3–4), and polymorphism of rs1554013, rs2243283, rs419598, rs4804800, rs2305619, and rs7248637 for recipients and rs7309123, rs419598, rs3921, rs4257674, and rs2305619 for donors were associated with increased proven and probable IFD after transplantation (P < 0.1). In multivariate analysis, the independent risk factors for IFD were grades 3–4 aGVHD, CMV reactivation, recipient rs2305619 and donor rs2305619 (PTX3; P < 0.05).

**Table 3:** Risk factors for occurrence of IFD

Variables	IFD (n = 22)	Univariate analysis		Multivariate analysis	
		χ^2^	P value	P value	HR (95%CI)
Recipient age			0.086	0.130	1.067 (1.014–1.122)
Recipient sex		0.604	0.437		
Male	14 (63.6%)				
Female	8 (36.4%)				
Underling disease		0.606	0.962		
AA	2 (9.1%)				
ALL	7 (31.8%)				
AML	10 (45.5%)				
MDS	2 (9.1%)				
Others	1 (4.5%)				
Donor age			0.6828		
> 36	13 (54.2%)				
≤ 36	11 (45.8%)				
Donor sex		0.491	0.483		
Male	18 (81.8%)				
Female	4 (18.2%)				
Blood type		1.747	0.417		
Match	11 (50.0%)				
Major mismatch	7 (31.8%)				
Minor mismatch	4 (18.2%)				
Neutrophil enengraftment	21 (95.5%)	2.292	0.130		
Platelet engraftment	19 (86.4%)	2.131	0.144		
aGVHD	21 (95.4%)				
I–II	16 (72.7%)	8.875	0.012	0.181	17.896 (2.145–49.361)
III–IV	5 (22.7%)	10.422	0.005	0.017	13.987 (1.132–62.999)
cGVHD	3 (13.6%)	1.686	0.430		
CMV	20 (90.9%)	6.962	0.008	0.007	35.97 (2.630–492.01)
Epstein–Barr virus	5 (22.7%)	1.128	0.298		
Recipient rs1554013		3.030	0.082	0.614	0.352 (0.078–1.589)
Recipient rs2243283		4.659	0.031	0.066	3.303 (0.924–11.809)
Recipient rs419598		3.080	0.079	0.088	4.133 (0.630–27.104)
Recipient rs4804800		3.522	0.061	0.073	5.898 (0.847–41.068)
Recipient rs2305619		4.520	0.034	0.006	10.249 (1.982–53.004)
Recipient rs7248637		4.159	0.041	0.749	1.933 (0.010–366.116)
Donor rs7309123		4.349	0.037	0.069	1.066 (0.256–4.449)
Donor rs419598		7.934	0.005	0.084	3.545 (0.900–13.965)
Donor rs3921		5.830	0.016	0.995	3.646 (0–1467)
Donor rs4257674		5.238	0.022	0.965	0.045 (0–1806)
Donor rs2305619		2.719	0.099	0.046	1.384 (0.351–5.466)

*AML* acute myeloid leukemia, *ALL* acute lympphocytic leukemia, *AA* aplastic anemia, *MDS* myelodysplastic syndrome, *HLA* human leukocyte antigen, *aGVHD* acute graft-versus-host disease, *cGVHD* chronic graft-versus-host disease, *CMV* cytomegalovirus

**Fig. 3:**
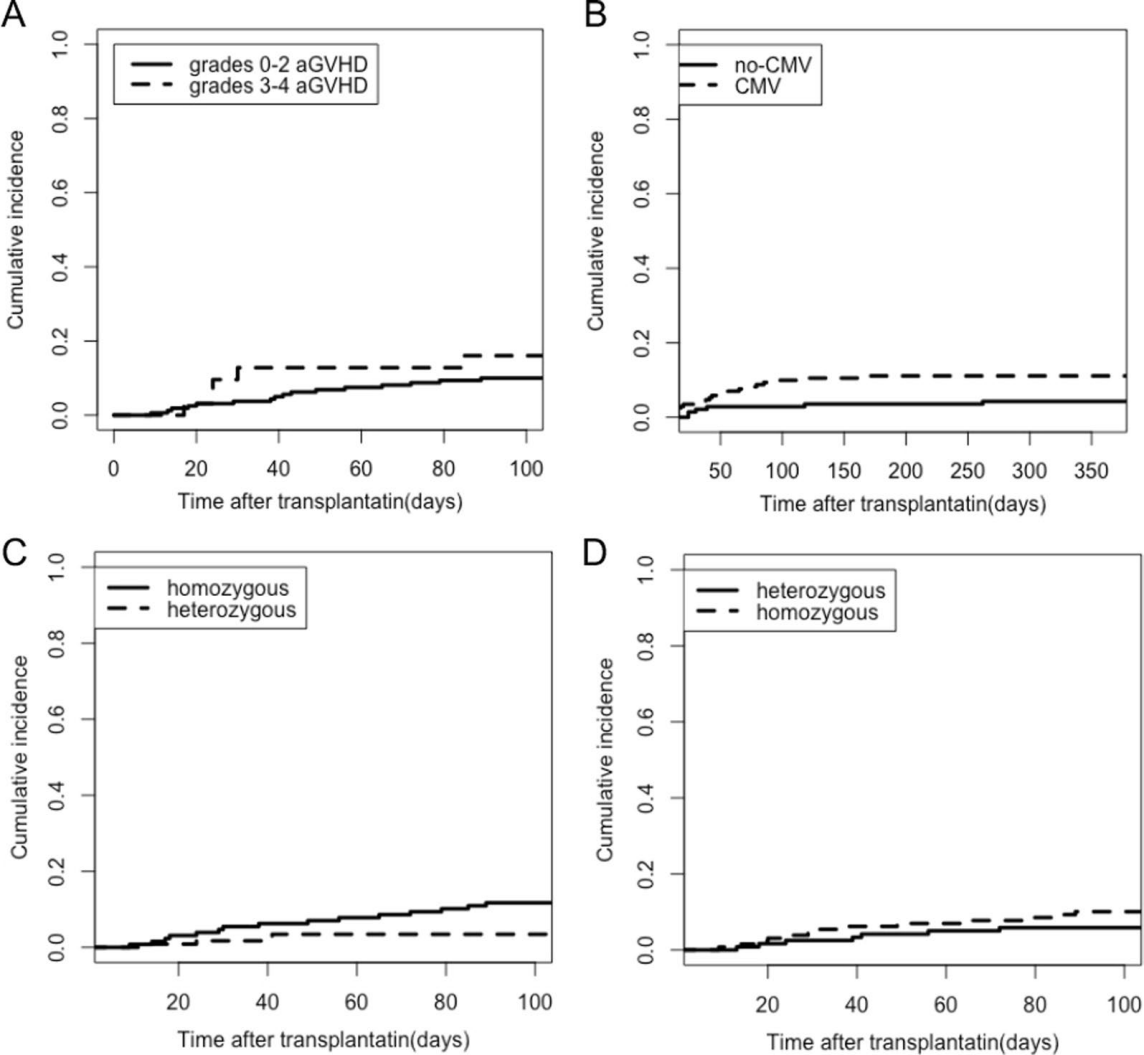
The effect of risk factors for IFD. Cumulative incidence of IFD in **A** grades 0–2 aGVHD and grades 3–4 aGVHD and **B** cytomegalovirus (CMV) reactivation compared to no-CMV. Cumulative incidence of IFD in heterozygous and homozygous for **C** PTX3 of the recipient and **D** PTX3 of the donor

Among the 22 IFD patients, 16 (72.7%) developed grades 0–2 and five (22.7%) developed grades 3–4 acute GVHD, and the cumulative incidence of IFD was significantly higher in grades 3–4 aGVHD than in grades 0–2 aGVHD at 30 days (12.8% versus 3.8%) and at 100 days (16.0% versus 10.0%; P = 0.009; Fig. 3A).

In all, 164 (65.3%) of 251 patients had cytomegalovirus reactivation, with a median time of 44 (10–270) days, and among IFD patients, 20 (90.9%) showed CMV reactivation. The cumulative incidence of IFD in CMV patients compared with no-CMV patients at 100 days and 1 year was 9.9% versus 2.8% and 11.1% versus 4.3%, respectively (P = 0.026; Fig. 3B).

### The impact of SNP on IFD

Forty-three SNPs of the recipient and 43 SNPs of the donor in the genomic DNA were genotyped. Recipient and donor rs2305619 (PTX3) were found to be significantly associated with susceptibility to IFD. In the overall IFD population, five patients (22.7%) were heterozygous (GA), 16 patients (72.7%) were homozygous (AA/GG) for PTX3 rs2305619 of the recipient, and one patient was missing data. The 30-day and 100-day cumulative incidence of IFD in homozygous and heterozygous patients was 5.4% versus 1.7% and 11.7% versus 3.4%, respectively (P = 0.035; Fig. 3C). Furthermore, seven donors (31.8%) were heterozygous (GA), and 15 donors (68.2%) were homozygous (GG/AA) for PTX3 rs2305619. The cumulative incidence in homozygous individuals was higher compared to heterozygous individuals at 30 days and at 100 days (5.4% versus 2.5% and 10.1% versus 5.9%; P = 0.046; Fig. 3D). However, there was no significant difference in PTX3 rs2305619 on OS, and the 1-year OS in homozygous and heterozygous patients and donors was 78.1% (95% CI 70.9–85.4%) and 81.2% (95% CI 74.1–88.3%; P = 0.301), 84.4% (95% CI 77.9–90.0%), and 74.4% (95% CI 66.6–82.2%; P = 0.100), respectively.

### Score system

**Fig. 4:**
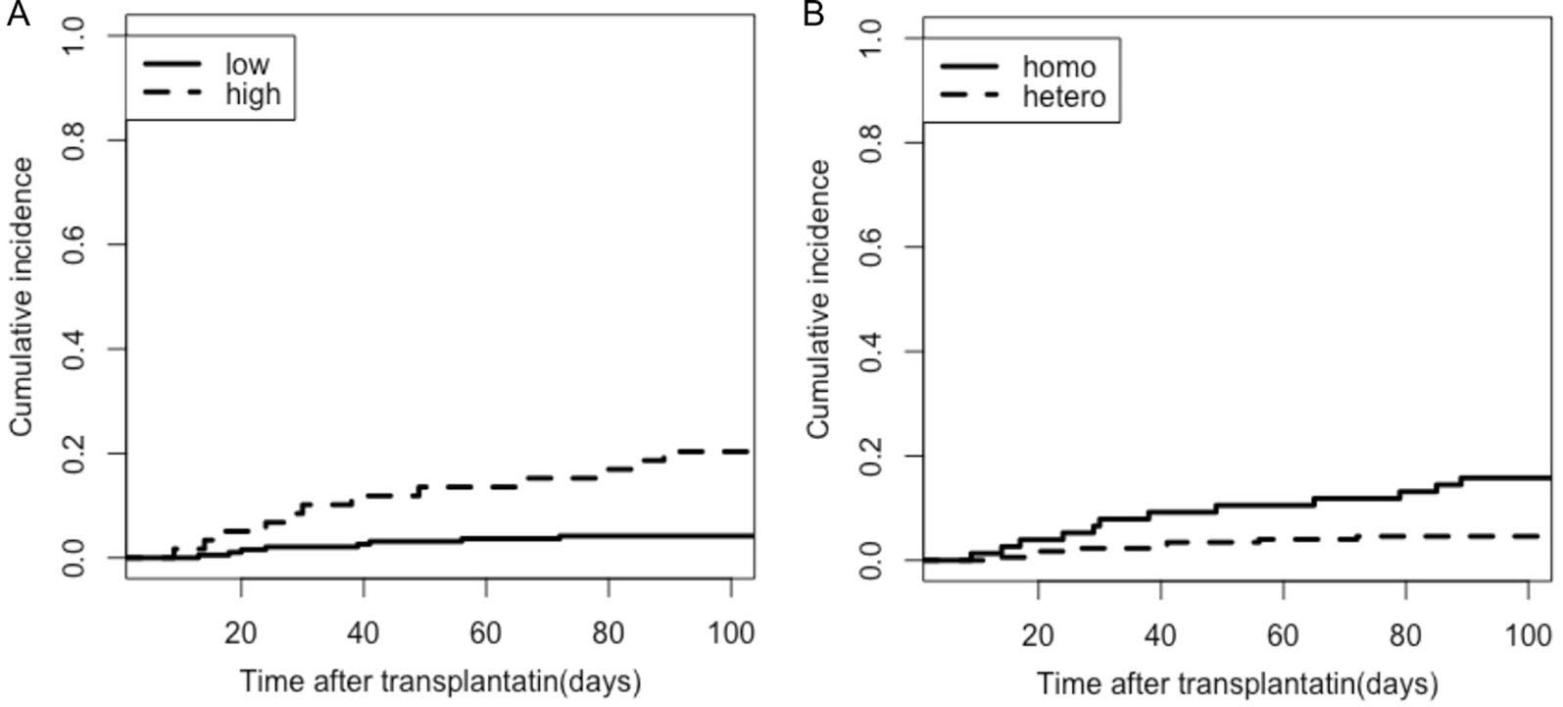
The cumulative incidence of IFD in **A** the scoring system for low and high groups. **B** Homozygous PTX3 of both recipients and donors (R+/D+) compared with heterozygous group (R−/D+, R+/D−, R−/D−)

Based on the independent predictors of IFD, we combined the variables (grades 3–4 aGVHD, CMV reactivation, PTX3 rs2305619 of recipients and donors) to develop the IFD scoring system to evaluate the incidence of IFD and stratified patients into low (0–2) and high (3–4) groups. The 30-day cumulative incidence of IFD in the low and high groups was 2.1% and 10.2%, and the 100-day cumulative incidence was 4.2% and 20.3%, respectively (P = 0.015; Fig. 4A). Additionally, we combined homozygous PTX3 rs2305619 for both recipients and donors (R+/D+) to compare the incidence of IFD with heterozygous group (R−/D+, R+/D−, R−/D−), and the 30-day and 100-day cumulative incidence of IFD were 7.9% versus 2.3% and 15.8% versus 4.6%, respectively (P = 0.01; Fig. 4B). Therefore, the scoring system might be useful in risk stratification of IFD after haplo-HSCT.

## Discussion and conclusion

Invasive fungal disease is a major cause of morbidity and mortality in patients following haplo-HSCT [17]. Recently, more than 20 SNPs have been shown to influence the risk of IFD in patients receiving HSCT; however, these studies have all focused on Caucasians and were not associated with clinical conditions [7, 18, 19]. In our study, we investigated whether 43 SNPs for recipients and donors in haplo-HSCT were associated with susceptibility to IFD in the Chinese Han population.

Genetic variations in genes regulated by the innate immune system play a pivotal role in the primary defense against infection and may influence the risk of IFD. Innate immunity includes physical barriers, cellular components, and soluble components (cytokines, chemokines, and others), which play an important role in the host response to various fungal pathogens [20]. Host cells express PRRs, such as DC-SIGN, toll-like receptors (TLRs), C-type lectin-like receptors (CLRs), and pentraxin-3 (PTX3) [21]. PTX3, which is produced by phagocytes and nonimmune cells at sites of inflammation or injury, has a nonredundant role in modulating various effector pathways involved in immune resistance to aspergillus, including activating innate immune cells and driving protective adaptive immunity [22]. PTX3 is stored in a ready-made form in mature polymorphonuclear leukocytes, and its secretion to extracellular traps promotes the control of fungal infection [23]. Given this, PTX3 deficiency may be associated with IFD following HSCT, which is a state of immunodeficiency and immune reconstitution.

We found that PTX3 rs2305619 in recipients and donors was correlated with an increased risk of developing IFD, while no significant difference was observed in OS. The highest risk of IFD was found for carriers of homozygous PTX3 rs2305619 (AA/GG). Additionally, patients carrying these genotypes showed an almost twofold increased risk, compared to those harboring heterozygous genotypes. Although it is now recognized that SNP in genes modulating immune response are likely to be risk factors of host susceptibility to fungal infections, thus far, few studies have shown differences in PTX3 in IFD patients who received HSCT. Among an Italian study with patients undergoing HSCT, Cristina Cunha et al. found that receipt of a transplant from a donor with a homozygous haplotype (h2/h2) in PTX3 was associated with a twofold increased risk of infection, compared to heterozygous groups (h1/h1, h1/h2), which was similar to the results seen in our study [11]. Moreover, they further elucidated that PTX3 deficiency in h2/h2 type affects the antifungal capacity of neutrophils, presumably due to messenger RNA instability, and this can be corrected after supplementing of exogenous PTX3. Tiantian Tang et al. conducted a case–control study to show that PTX3 rs2305619 had no genotypic distribution differences, but rs3816527 was associated with invasive pulmonary aspergillosis (IPA) in non-neutropenic patients [24]. However, it is difficult to directly compare our data, and several points should be taken into consideration. Although there are studies demonstrated that PTX3 levels was associated with aGVHD severity [25], no evidence has been reported to show the association between PTX3 polymorphism and aGVHD [26, 27]. And our current study didn’t find the association between PTX3 polymorphism and aGVHD. Although neutropenia is a significant risk factor for opportunistic fungal infection, in our study, we confirmed that grades 3–4 aGVHD and CMV reactivation were risk factors of IFD, but neutropenia also led to immune deficiency. Moreover, eight of the 22 patients in our study had a defined etiology for IFD, and the majority were molds (aspergillus, mucor), which may lead to different results. Additionally, the previous study may have differed from our study in terms of ethnic differences, patients’ age, transplant protocol, and other factors. Further multicenter study is warranted to confirm the effect of PTX3 on IFD.

Recently, many studies demonstrated that Dectin-1, TLR4, IL10, and so on are associated with IFD in AML patients or donors following HSCT [10, 19, 28, 29]. These findings, however, could not be confirmed in our data. The above SNPs were involved in our data, but no differences were observed in our results. Further studies are warranted to confirm the effect on IFD in HSCT patients.

Several lines of evidence suggest the relevance of SNP effects in IFD following transplantation; however, up to now, no studies have combined the effects with clinical factors. Similar to previous studies, our study demonstrated that severe aGVHD and CMV reactivation have significant differences in IFD, compared to HLA-matched donors, prolonged neutropenia, cGVHD, and so on [3, 30]. We acknowledged these similarities and differences, and further studies are warranted to confirm this. In this study, we first assessed the risk factors of IFD, including SNPs and clinical conditions, and then further developed a predictive scoring system for IFD following HSCT. These results were promising and showed that patients in the high-risk group experienced a higher risk of IFD. Interestingly, we also evaluated the incidence of IFD in the recipient and donor PTX3 rs2305619 of both homozygous type, which even higher than heterozygous type, and considering this encouraging result, we concluded that homozygous PTX3 rs2305619 increased the susceptibility to IFD. These results show that the scoring system can be used for counseling patients and providing a benchmark for further interventions.

There are some limitations to our present study. This was a single-center prospective study, the number of IFD patients was small, and IFD categories were not classified. In addition, the PTX3 rs2305619 polymorphisms were found to be significantly associated with invasive fungal disease but invasive aspergillosis in hematopoietic-cell transplants. Third, this study includes both adult and pediatrics that may have different characteristics in IFD. Although we have the same results while we performed sub-group analysis in only adults, further validation in pediatrics might need further investigation. Additionally, further studies using large cohorts are necessary to clarify the mechanism.

In conclusion, our study demonstrates that the PTX3 rs2305619 increase susceptibility to IFD after haplo-HSCT in a Chinese Han population, although no clear pathophysiological mechanism can be drawn from our data. Further studies are needed to elucidate the exact role of SNPs in susceptibility to IFD in HSCT patients. Furthermore, we developed a scoring system for IFD patients who received HSCT. For future studies, we plan to validate this system using an independent dataset for patients receiving HSCT.

## Supplementary Information

**Additional file 1: Table S1.** The PRRs and whose SNPs.

## Data Availability

The data used to support the findings of this study are available from the corresponding author upon request.

## Abbreviations

IFD: Invasive fungal disease
haplo-HSCT: Haploidentical stem cell transplantation
HLA: Human leukocyte antigen
GVHD: Graft-versus-host disease
CMV: Cytomegalovirus
SNPs: Single nucleotide polymorphisms
PTX3: Pentraxin-3
G: β-(1–3)-Glucans
GM: Galactomannans
OS: Overall survival
DFS: Disease free survival
TRM: Treatment-related mortality
CIR: Cumulative incidence of relapse
CR: Complete response
EORTC/MSG: European Organization for Research and Treatment of Cancer and the Mycoses Study Group and Research Consortium
aGVHD: Acute graft-versus-host disease
cGVHD: Chronic graft-versus-host disease
TLRs: Toll-like receptors
CLRs: C-type lectin-like receptors
IPA: Invasive pulmonary aspergillosis
