# Supplementary material for: Recipient and donor PTX3 rs2305619 polymorphisms increase the susceptibility to invasive fungal disease following haploidentical stem cell transplantation: a prospective study
Source: BMC Infect Dis. 2022 Mar 26;22:292. doi: 10.1186/s12879-022-07298-2 (PMC8962575; doi:10.1186/s12879-022-07298-2)
Supplement: Supplementary file 1 — Additional file 1: Table S1. The PRRs and whose SNPs. [file 12879_2022_7298_MOESM1_ESM.docx]

Table S1 The PRRs and whose SNPs

| PRRs | SNPs | PRRs | SNPs |
| --- | --- | --- | --- |
| TLR1 | rs4833095 | IFN-$\gamma$ | rs2069705 |
|  | rs5743611 | TNF-$\partial$ | rs1800629 |
| TLR3 | rs3775296 | IL4 | rs1889570 |
| TLR4 | rs4986790 |  | rs2070874 |
|  | rs4986791 |  | rs2227284 |
| TLR6 | rs5743810 |  | rs2243248 |
| TLR5 | rs5744168 |  | rs2243288 |
| TLR9 | rs352140 |  | rs2243292 |
| DC-SIGN | rs1465384 |  | rs3804513 |
|  | rs4804800 |  | rs2243247 |
|  | rs2287886 |  | rs2243250 |
|  | rs7248637 |  | rs2243283 |
| PTX3 | rs1840680 | IL-17A | rs2275913 |
|  | rs3816527 | IL-17F | Rs763780 |
|  | rs2305619 | IL-1RN | Rs419598 |
| Dectin-1 | rs16910526 | IL-1B | Rs16944 |
|  | rs3901533 | IL-6 | Rs1800797 |
|  | rs7309123 | IL-23 | Rs11209026 |
| MBL2 | rs1800450 | C-X-C-10 | Rs15543013 |
| IL10 | rs1800871 |  | Rs3921 |
|  | rs1800872 |  | Rs4257674 |
|  | rs1800896 |  |  |

PRRs pattern recognition receptors, SNPs single nucleotide polymorphisms, TLR toll-like receptor, DC-SIGN Dendritic Cell-Specific Intercellular adhesion molecule-3-Grabbing Non-integrin, PTX3 pentraxin-3, Dectin-1 Dendritic cell-associated C-type lectin-1, MBL2 mannose-binding lectin, IL interleukin, IFN interferon, C-X-C-10 Chemokine ligand 10
